# Case report of homozygous deletion involving the first coding exons of *GCNT2* isoforms *A* and *B* and part of the upstream region of *TFAP2A* in congenital cataract

**DOI:** 10.1186/s12881-016-0316-0

**Published:** 2016-09-08

**Authors:** Hannah Happ, Eric Weh, Deborah Costakos, Linda M. Reis, Elena V. Semina

**Affiliations:** 1 Department of Pediatrics and Children’s Research Institute, Medical College of Wisconsin, Milwaukee, WI 53226 USA; 2 Department of Ophthalmology, Medical College of Wisconsin, Milwaukee, WI 53226 USA; 3 Department of Cell Biology, Neurobiology & Anatomy, Medical College of Wisconsin, Milwaukee, WI 53226 USA

**Keywords:** *GCNT2* deletion, Congenital cataract, Case report

## Abstract

**Background:**

Congenital cataracts affect 3–6 per 10,000 live births and represent one of the leading causes of blindness in children. Congenital cataracts have a strong genetic component with high heterogeneity and variability.

**Case presentation:**

Analysis of whole exome sequencing data in a patient affected with congenital cataracts identified a pathogenic deletion which was further defined by other techniques. A ~98-kb homozygous deletion of 6p24.3 involving the first three exons (two non-coding and one coding) of *GCNT2* isoform A, the first exon (coding) of *GCNT2* isoform B, and part of the intergenic region between *GCNT2* and *TFAP2A* was identified in the patient and her brother while both parents were found to be heterozygous carriers of the deletion. The exact breakpoints were identified and revealed the presence of Alu elements at both sides of the deletion, thus indicating Alu-mediated non-homologous end-joining as the most plausible mechanism for this rearrangement. Recessive mutations in *GCNT2* are known to cause an adult i blood group phenotype with congenital cataracts in some cases. The *GCNT2* gene has three differentially expressed transcripts, with *GCNT2B* being the only isoform associated with lens function and *GCNT2C* being the only isoform expressed in red blood cells based on earlier studies; previously reported mutations/deletions have either affected all three isoforms (causing blood group and cataract phenotype) or the C isoform only (causing blood group phenotype only). Dominant mutations in *TFAP2A* are associated with syndromic anophthalmia/microphthalmia and other ocular phenotypes as part of Branchio-Ocular-Facial-Syndrome (BOFS). While the patients do not fit a diagnosis of BOFS, one sibling demonstrates mild overlap with the phenotypic spectrum, and therefore an effect of this deletion on the function of *TFAP2A* cannot be ruled out.

**Conclusions:**

To the best of our knowledge, this is the first case reported in which disruption of the *GCNT2* gene does not involve the C isoform. The congenital cataracts phenotype in the affected patients is consistent with the previously defined isoform-specific roles of this gene. The *GCNT2-TFAP2A* region may be prone to rearrangements through Alu-mediated non-homologous end-joining.

**Electronic supplementary material:**

The online version of this article (doi:10.1186/s12881-016-0316-0) contains supplementary material, which is available to authorized users.

## Background

Congenital cataracts are diagnosed within the first year of life. These cataracts are one of the leading causes of blindness in children and are estimated to occur with a prevalence of 3–6 per 10,000 live births [1]. Congenital cataracts may appear either in isolation or in association with other ocular or systemic anomalies. Up to 25 % of congenital cataracts are thought to be caused by genetic defects [2]. The genetic landscape of mutations causing congenital cataract is extremely diverse; more than 40 genes and additional loci have been associated with nonsyndromic cataract [2–6].

*GCNT2* (glucosaminyl (N-acetyl) transferase 2, I-branching enzyme) was first identified in 2001 as the gene encoding for the glycosyltransferase responsible for the human blood group I antigen. Recessive mutations in *GCNT2* result in an adult i blood group phenotype, which is also associated with congenital cataracts in some cases [7]. Alternative splicing of the *GCNT2* gene produces three transcripts (A, B, and C). The three transcripts share a common second and third coding exon with a unique first exon for each isoform; differing expression profiles were identified for the transcripts with only the *GCNT2B* isoform expressed in lens epithelial cells and only the *GCNT2C* isoform expressed in reticulocytes [8]. To date, seven missense mutations, one nonsense mutation, and two large deletions have been reported; mutations in exon 1C, affecting only the *GCNT2C* isoform, cause the adult i blood group without cataracts while mutations/deletions affecting exons 2 and 3, shared by all isoforms, result in the adult i blood group along with congenital cataract [7–11].

## Case presentation

**Fig. 1:**
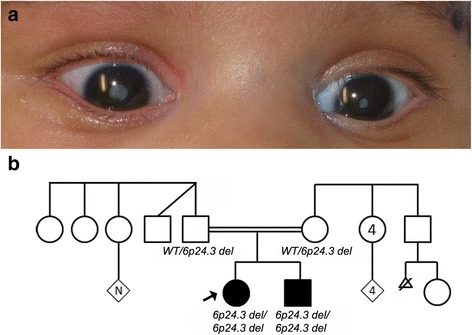
Patient photographs and pedigree. **a** Photograph of Patient 1’s eyes at 2 months of age showing bilateral cataract. **b** Pedigree showing both affected siblings with a homozygous deletion of 6p24.3 while the unaffected parents are heterozygous carriers. WT: wild type; black arrow indicates proband

Patient 1(individual II:1) is an 18-month old Pakistani female affected with bilateral dense central congenital cataract (Fig. 1a, Table 1) which were visually significant and required extraction at 2 months of age, mild asymmetry of the palpebral fissures, and left nasolacrimal duct obstruction; her development is normal and growth parameters are generally normal with the exception of borderline microcephaly (length 83.8 cm, 75–90th centile; weight 10.2 kg, 25–50th centile; and head circumference 44 cm (3rd centile)). Physical exam at 4 months of age identified hypotelorism (familial) and mildly widely-spaced nipples. Her younger brother, age 6 months, was similarly affected with visually significant bilateral dense central congenital cataracts requiring extraction around 2 months of age; his length (67.5 cm, 25–50th centile), weight (6.8 cm, 5–10th centile), and head circumference (42.5 cm, 10–25th centile) are all within the normal range (Table 1). Family history shows unaffected second-cousin parents with additional endogamous mating within the family. A double second-cousin to the proband is affected with bilateral non-syndromic anophthalmia/ microphthalmia with no additional details available.

**Table 1:** Phenotype and genotype information of the affected patients

Patient	Lens phenotype	Other features	Development	Deletion	Genes involved
Patient 1	Bilateral dense central congenital cataracts; extraction at ~2 months of age	Borderline microcephaly (3rd centile), mild asymmetry of the palpebral fissures, left nasolacrimal duct obstruction, hypotelorism, somewhat widely-spaced nipples	WNL	97.9 kb homozygous deletion of 6p24.3	The first coding exons of *GCNT2A* and *GCNT2B,* two 5’noncoding exons of *GCNT2A*, and a part of the region upstream of *TFAP2A*
Patient 2	Bilateral dense central congenital cataracts; extraction at ~2 months of age	None	WNL	97.9 kb homozygous deletion of 6p24.3	The first coding exons of *GCNT2A* and *GCNT2B,* two 5’noncoding exons of *GCNT2A*, and a part of the region upstream of *TFAP2A*

## Materials and methods

Whole exome sequencing was performed by Macrogen (previously Axeq) and analyzed as previously described [12]; briefly, exome data from the proband was analyzed using the SNP & Variation Suite (SVS; Golden Helix, Bozeman, MT, USA) to identify/exclude mutations in the coding and splicing regions of 40 known nonsyndromic cataract genes and 7 additional crystallins [3–6]; synonymous variants and variants with a frequency of >1 % in the general population (http://exac.broadinstitute.org, http://evs.gs.washington.edu/EVS/, http://www.1000genomes.org/) were considered to be benign variants. Copy number variation analysis was completed by screening exome sequencing data using the Copy Number Inference From Exome Reads (CoNIFER) v0.2.2 software package as previously outlined [13]; regions of interest were further verified by independent quantitative PCR reactions using DNA samples from the proband and other available familial samples with SYBR Green PCR Master Mix (Applied Biosystems/Life Technologies, Carlsbad, CA, USA). qPCR reactions utilized three region-specific probes (Additional file 1: Table S1) and were performed as follows: primers located within regions of interest were designed using Primer3Plus software (http://sourceforge.net/projects/primer3/) using qPCR settings. Each reaction was comprised of five nanograms of DNA in a total reaction volume of 12uL. Each primer set was run three times in triplicate using patient, parental or control DNA on a Bio-Rad CFX Connect Real-Time PCR machine (Bio-Rad, Hercules, CA, USA). A primer set for the housekeeping gene *RPPH1* (ribonuclease P RNA component H1) was used to normalize all data. A probe located in *NDP* (Norrie disease (pseudoglioma)), located on the X-chromosome, was used as a copy-loss control. All experiments included a no-template control and an unaffected human DNA sample with presumably normal copy number at each region for comparison. Copy number changes were calculated using the 2^-ΔΔCt^ method as previously described [14]. Following qPCR confirmation, the size and exact breakpoints of the deletion were determined using a series of regular PCR reactions that utilized primers located on both ends of the region (as defined by CoNIFER and qPCR analysis) and standard conditions (Additional file 1: Table S1). Since the patients were apparently homozygous for the deletion, no amplification product indicated that the primer(s) are located inside of the deleted region while the presence of a PCR product indicated primers outside of the deletion. Once sequences bordering the deleted region on the centromeric and telomeric sides were determined, the corresponding primers were used to amplify a 1.5 kb region across the breakpoints. The resultant product was cloned into pCRII-TOPO® (Life Technologies, Carlsbad, CA, USA) vector using the manufacturer’s protocols and sequenced bidirectionally with M13 forward and reverse primers using Big Dye Terminator v3 chemistry and an ABI 3730XL sequencer (Applied Biosystems/Life Technologies, Carlsbad, CA, USA); the obtained sequences were compared with the corresponding reference sequence using BLAST (http://blast.ncbi.nlm.nih.gov/Blast.cgi).

## Results and discussion

**Fig. 2:**
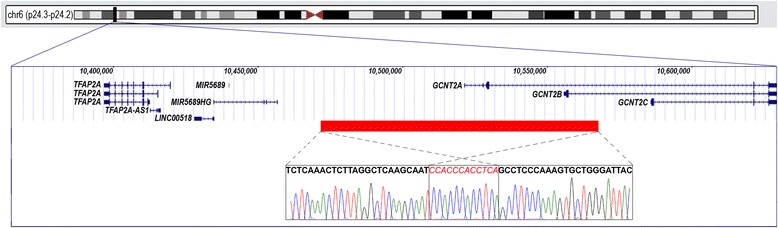
Schematic presentation of the chromosome 6p24.3−24.2 region and the identified deletion. The UCSC Genome Browser (http://genome.ucsc.edu) view of the deleted region indicating the positions of genes is included; the deletion identified in the affected family is shown as a rectangular red box; the DNA sequence across the breakpoint for the deleted allele is shown at the bottom of the drawing with regions corresponding to the telomeric and centromeric flanks of the deletion indicated by dashed lines and a 12-nt repeat highlighted in red font

Review of the whole exome sequencing (WES) data from Patient 1 did not identify any potentially pathogenic variants (with only two synonymous variants) in known nonsyndromic cataract genes. The WES data was then analyzed for copy number variation which revealed a potential 208-kb deletion (6p24.3 chr6: 10,412,788-10,621,660) affecting *TFAP2A* and *GCNT2*. The deletion was verified using qPCR probes located in the first coding exon of *GCNT2* isoform A and the first exon of *TFAP2A*; the qPCR confirmed deletion of the *GCNT2* sequence in both unaffected parents (haploid, heterozygous) and affected children (complete loss, homozygous) while diploid copy of the *TFAP2A* sequence was identified in all family members. Further analysis of the region by a series of regular PCR reactions using affected DNA identified the centromeric breakpoint between chr6:10472330–10472606 (set 7; diploid) and chr6:10474759–10474901 (set 8; complete loss) and the telomeric breakpoint between chr6:10570580–10570905 (set 13, complete loss) and chr6:10571951–10572257 (set 14, diploid). Primer sets designed to span the deleted region produced a ~1.5 kb product from the DNA of the affected patients. Sequencing of this product identified the exact deletion breakpoint sites: their analysis revealed the presence of Alu repeats and specifically a 12-bp identical sequence at both sides of the deleted region of 97.974-kb (hg19, chr6: 10,473,864–10,571,838) (Fig. 2). The homozygous deletion encompassed four exons of *GCNT2* (the first two noncoding and one coding exons of isoform A and the first coding exon of isoform B) and extended 47.471-kb upstream of the most 5’ exon of the *GCNT2* gene (Fig. 2). The distance from the telomeric end of the deletion to the nearest protein-coding gene, *TFAP2A* (transcription factor AP-2 alpha), is 54.300-kb. The distance from the centromeric end of the deletion to the fist exon of *GCNT2* isoform C is 13.922-kb. Although *GCNT2C* and *TFAP2A* were not included in the deletion, effects on their expression through possible interference with regulatory elements cannot be ruled out.

Genomic deletions of *GCNT2* have been previously reported in two families with blood group i and congenital cataracts but both deletions included exons 2 and 3 which are shared by all isoforms [7, 11]. Borck and colleagues noted that the *GCNT2* locus is rich with Alu elements and therefore is likely a hotspot for deletions or duplications to occur [11]. The *GCNT2* gene has three differentially expressed transcripts, with *GCNT2B* being the only isoform associated with lens function and *GCNT2C* being the only isoform expressed in red blood cells [8]. The GCNT2 protein modifies the i antigen, a linear sphingoglycolipid present on the cell surface of most human cells as well as on glycoproteins in body fluids, into the active branched I antigen; the i/I antigens are thought to play a role in the regulation of cell growth and differentiation in the developing lens [8, 9].

The deletion described in this case report differs from previously reported deletions and mutations since it only affects the *GCNT2A* and *GCNT2B* isoforms and leaves the *GCNT2C* isoform intact. Previous studies demonstrated that only the *GCNT2B* isoform is expressed in lens epithelial cells and patients with mutations which specifically affect the C isoform demonstrate the adult i phenotype without congenital cataracts [8]. Thus, the presence of cataract in the affected patients reported here with a clear disruption of *GCNT2A* and *B* isoforms only is consistent with the isoform-specific roles identified for this gene. Additionally, in the case reported here we were able to identify the exact sequences at the breakpoints and clearly implicate Alu-mediated non-homologous end-joining as a mechanism for this rearrangement. This mechanism has been previously reported by our and other groups [15, 16]).

The deletion reported here also extends into the genomic region upstream of *GCNT2* and *TFAP2A* which are positioned in a head-to-head orientation. *TFAP2A* is approximately 100-kb distal to *GCNT2* and the deletion removes approximately 47-kb of genomic sequence between the two genes. It is possible that this deletion could affect *TFAP2A* function through removal/rearrangement of regulatory elements, as has been shown for other genes [17–19]. *TFAP2A* is a retinoic acid responsive transcription factor which is required for normal development of the lens and optic cup as well as for parts of the craniofacial region. Heterozygous mutations in *TFAP2A* cause Branchio-Ocular-Facial-Syndrome (BOFS) characterized by craniofacial phenotypes (distinct facial features, microcephaly, and cleft lip/palate), skin defects in the cervical region or regions around the ear, ocular defects (microphthalmia, coloboma, strabismus, cataract, or ptosis), lacrimal duct obstruction, and hearing loss [20–22]. Missense mutations account for the majority of *TFAP2A* variants, however whole gene deletions have also been reported. To date, no deletions affecting the upstream region of *TFAP2A,* but not the coding region itself, have been reported. Careful physical examination of the patients did not identify sufficient features to warrant a diagnosis of BOFS in the siblings, but Patient 1 did show borderline microcephaly, mild asymmetry of the palpebral fissures, left nasolacrimal duct obstruction, and somewhat widely spaced nipples. While the shared cataract phenotype observed in the affected siblings is consistent with the *GCNT2* deficiency alone, an effect of this deletion on the function of *TFAP2A* and the observed phenotypes cannot be completely ruled out. Interestingly, a double second-cousin to the proband has been reported to be affected with bilateral anophthalmia/ microphthalmia (A/M), an ocular condition that is more consistent with the *TFAP2A* spectrum. It is possible that the familial deletion expanded to include *TFAP2A* in this patient; alternatively, the A/M diagnosis may have an independent genetic etiology. Unfortunately, no other familial samples were available for further study.

## Conclusions

We identified a ~98-kb homozygous deletion involving several exons of *GCNT2* and the region upstream of *TFAP2A* in two children affected with congenital cataracts from a consanguineous family of Pakistani decent. This cataract-causing deletion removes the first coding exons of *GCNT2* isoforms *A* and *B* but leaves the *GCNT2C* sequence intact, providing further support for the isoform-specific roles of this gene; this is the first disruption of *GCNT2* reported which does not affect isoform *C*. While the patients do not fit a diagnosis of BOFS, one sibling demonstrates mild overlap with the phenotypic spectrum, and therefore an effect of this deletion on the function of *TFAP2A* cannot be ruled out.

## Additional file

Additional file 1: Table S1. Summary of PCR/qPCR reactions and copy number status in the affected family. (DOCX 21 kb)

## Abbreviations

A/M: Anophthalmia/Microphthalmia
BOFS: Branchio-ocular-facial-syndrome
GCNT2: Glucosaminyl (N-acetyl) transferase 2, I-branching enzyme
TFAP2A: Transcription factor AP-2 alpha
WES: Whole exome sequencing
