# Supplementary material for: Case report of homozygous deletion involving the first coding exons of GCNT2 isoforms A and B and part of the upstream region of TFAP2A in congenital cataract
Source: BMC Med Genet. 2016 Sep 8;17(1):64. doi: 10.1186/s12881-016-0316-0 (PMC5016880; doi:10.1186/s12881-016-0316-0)
Supplement: Additional file 1: Table S1. — Summary of PCR/qPCR reactions and copy number status in the affected family. (DOCX 21 kb) [file 12881_2016_316_MOESM1_ESM.docx]

**Supplemental Table 1.** Summary of PCR/qPCR reactions and copy number status in the affected family.

| **Name** | **set** | **Forward primer** | **Reverse primer** | **Method** | **Product size (bp)** | **Genomic coordinates of amplified region** | **Copy number** | |
| --- | --- | --- | --- | --- | --- | --- | --- | --- |
|  |  |  |  |  |  |  | **Proband and**  **Affected sib** | **Unaffected parents** |
| TFAP2A_delcheck2f | 1 | TTTCGCAGCTGGTTGCAAG | TACGAGGACTGCGAGGTAAG | qPCR | 108 | chr6:10415081-10415188 | 2 | 2 |
| LINC00518_dc2 | 2 | AAATATGGGTGCAGCATGGG | TTGGTGGAAGAGCAAAGTGC | qPCR | 117 | [chr6:10431509-10431625](http://genome.ucsc.edu/cgi-bin/hgTracks?hgsid=438226031_jZovBuaOfjvT01VaLbqaRTS4AO63&db=hg19&position=chr6:10431509-10431625&hgPcrResult=pack) | 2 | 2 |
| GCNT2_delcheck2 | 3 | GCAAGACTTTCCCCTGAAAACC | ACTCCGGGGGTGATATTTTTCC | qPCR | 81 | chr6:10529720-10529800 | 0 | 1 |
|  | | | | | | | | |
| Chr6_delcheck1 | 4 | CAGAAGGCCTGAGTCCCATG | AGGCAAAATAGGCTCGTGCT | PCR | 468 | chr6:10450740-10451207 | 2 | 2 |
| Chr6_delcheck2 | 5 | GTTTTGCTGCCTTGGTCCTG | TGCTGTGCCTGAGGAACAAT | PCR | 406 | chr6:10460347-10460752 | 2 | 2 |
| Chr6_Delcheck7 | 6 | TGAATGAGGGGAGGAGGGAG | GGGCTGATTCGACTCCACAA | PCR | 401 | chr6:10465933-10466333 | 2 | 2 |
| Chr6_Delcheck8 | 7 | GGCCAAAGCAAACAACAAAAGG | TGCAGCCTCTTTCATGCTCT | PCR | 277 | chr6:10472330-10472606 | 2 | 2 |
| Chr6_Delcheck9 | 8 | TTTCCTTGGGCTGCATCTGG | ACTGCATGCACATTTCCAACT | PCR | 143 | chr6:10474759-10474901 | 0 | 1 |
| Chr6_delcheck3 | 9 | CCTGCTGATCCCGACCATAC | ACAGGGGTCAGGGAGGTAAT | PCR | 400 | chr6:10478528-10478927 | 0 | 1 |
| Chr6_Delcheck10 | 10 | GCCTTGGCTGTGATCTCCAT | CAGCTTCACATGGCTCAGGA | PCR | 403 | chr6:10545512-10545914 | 0 | 1 |
| Chr6_delcheck11 | 11 | CACAGTGACAGGATGGCCTT | ACGGGAGTCCAGATGAGTGA | PCR | 395 | chr6:10566099-10566493 | 0 | 1 |
| Chr6_delcheck13 | 12 | AGGATCCAGCCAGCACATTC | GGCAGGATTACAGGCATGGT | PCR | 430 | chr6:10568590-10569019 | 0 | 1 |
| Chr6_delcheck14 | 13 | GATGGGAGTTGGGCTTTCGA | AGGAGCTCAGACTTGGACCA | PCR | 326 | chr6:10570580-10570905 | 0 | 1 |
| Chr6_delcheck20 | 14 | TCCACTGGAAAACATCTGTGT | GCATCAGTCCTAACCACTCCC | PCR | 307 | chr6:10571951-10572257 | 2 | 2 |
| Chr6_delcheck15 | 15 | GCTCTGTGGGAAAGGAGCTA | TCTGGTGTTAACTGCCCAGC | PCR | 424 | chr6:10573219-10573642 | 2 | 2 |
| Chr6_delcheck16 | 16 | TGAACCCAGGCACCTTTCTC | GGAAAAGAGGAGAGGCACCC | PCR | 365 | chr6:10575082-10575446 | 2 | 2 |
| Chr6_delcheck17 | 17 | GCCTGAAGTAGAATCACATGGC | GCTCCTGCTTACAACACTTTT | PCR | 374 | chr6:10576635-10577008 | 2 | 2 |
| Chr6_delcheck18 | 18 | CAAAACTGTTGTCCCAATCTATTATTT | ATGTCTATGAAAGGACTTGAAAGCTG | PCR | 250 | chr6:10579459-10579708 | 2 | 2 |
| Chr6_delcheck19 | 19 | TTTTGGATACTGCTGTTTTGAACT | TCACACACACTCTAGAAATGTGACT | PCR | 360 | chr6:10581815-10582174 | 2 | 2 |
| Chr6_delcheck12 | 20 | CTGTTCCAACTGGCACAAGC | CAACCCCCTCTCCAACTTCC | PCR | 362 | chr6:10583580-10583941 | 2 | 2 |
| Chr6_delcheck4 | 21 | TGAACTGCTCTGAGCCAAGG | TCCCCCTCGTTACCAAGCTA | PCR | 369 | chr6:10610726-10611094 | 2 | 2 |
| Chr6_delcheck5 | 22 | GCACCTGAGAAGAGGAACCG | TCTCCAACACCCCACAATGG | PCR | 444 | chr6:10629343-10629786 | 2 | 2 |
| Chr6_delcheck6 | 23 | ACCTGTGGCCTGGAAAAGAG | GATTGCCCAGACCTCTGACC | PCR | 386 | chr6:10652917-10653302 | 2 | 2 |
